# Pancreatic Ductal Adenocarcinoma After Hepatitis C Infection

**DOI:** 10.1001/jamanetworkopen.2025.43701

**Published:** 2025-11-14

**Authors:** Rachel N. Levinson, Ryan Bushman, Janet P. Tate, Melissa Skanderson, Catherine Mezzacappa, Lesley S. Park, Cynthia A. Brandt, Kevin M. Schuster, Gyanprakash A. Ketwaroo, Yu-Xiao Yang, Amy C. Justice, Louise L. Wang

**Affiliations:** 1 Section of Digestive Diseases, Department of Internal Medicine, Yale School of Medicine, New Haven, Connecticut; 2 VA Connecticut Healthcare System, West Haven, Connecticut; 3 Section of General Medicine, Department of Internal Medicine, Yale School of Medicine, New Haven, Connecticut; 4 Department of Epidemiology & Population Health, Stanford University School of Medicine, Palo Alto, California; 5 Department of Biomedical Informatics and Data Science, Yale School of Medicine, New Haven, Connecticut; 6 Department of Surgery, Yale School of Medicine, New Haven, Connecticut; 7 Division of Gastroenterology, Perelman School of Medicine, University of Pennsylvania, Philadelphia, Pennsylvania; 8 Corporal Michael J. Crescenz VA Medical Center, Philadelphia, Pennsylvania; 9 School of Public Health, Yale University, New Haven, Connecticut

## Abstract

**Importance:**

Although hepatitis C virus (HCV) is an oncovirus, its association with the risk of pancreatic ductal adenocarcinoma (PDAC) is unclear. In addition, it is unknown whether there is differential risk for PDAC across HCV genotypes.

**Objective:**

To assess the association between chronic HCV and PDAC.

**Design, Setting, and Participants:**

This retrospective, national, population-based cohort study was conducted across Veterans Health Administration (VA) sites. The study included veterans with HCV testing documented in the VA or VA-linked Medicare with at least 1 inpatient or outpatient visit between October 1, 2001, and September 30, 2020. Patients were followed-up for at least 18 months after this visit. Data were analyzed from October 2023 to September 2025.

**Exposure:**

HCV status was categorized as chronic HCV, exposure to HCV, or no chronic HCV infection.

**Main Outcomes and Measures:**

The association of HCV status with PDAC was evaluated using Cox proportional hazards regression, adjusting for demographic and clinical confounders. Analysis was substratified by HCV genotype.

**Results:**

Of 6 330 856 people tested for HCV (5 841 571 men [92.3%]; median [IQR] age, 61.6 years [49.9-70.1]), 246 218 (3.9%) had chronic HCV and 209 492 (3.3%) were exposed. Of the 33 451 individuals (0.5%) who developed PDAC, age at diagnosis was younger among those with vs those without HCV (median [IQR] age, 65.0 [59.9-69.6] years vs 72.4 [66.7-79.0] years). Compared with no HCV infection, chronic HCV infection (adjusted hazard ratio [aHR], 1.76; 95% CI, 1.67-1.86) and HCV exposure (aHR, 1.18; 95% CI; 1.11-1.25) were associated with increased risk of incident PDAC. Hazards for PDAC were greater for HCV genotype 3 (aHR, 2.02; 95% CI, 1.67-2.45) and genotype 1 (aHR, 1.75; 95% CI, 1.64-1.87) than for genotype 2 (aHR, 1.35; 95% CI, 1.14-1.60) compared with no HCV infection.

**Conclusions and Relevance:**

In this cohort study of veterans, chronic HCV infection was associated with a 1.8-fold higher risk of PDAC diagnosis, and HCV genotypes 3 and 1 had greater PDAC risk than genotype 2. These findings prompt future research on the mechanisms underlying this association and the impact of HCV treatment on PDAC risk.

## Key Points

**Question:**

Is there an association between chronic hepatitis C virus (HCV) infection and pancreatic cancer, independent of other risk factors?

**Findings:**

In this cohort study of 6.3 million veterans, individuals with chronic HCV developed pancreatic cancer at younger ages and had an increased risk of pancreatic cancer compared with individuals without HCV infection. Risk for pancreatic cancer also varied by HCV genotype.

**Meaning:**

These findings suggest that chronic HCV is a potentially modifiable risk factor for pancreatic cancer.

## Teaser

This cohort study assesses the association between chronic hepatitis C virus infection and pancreatic ductal adenocarcinoma.

## Introduction

Pancreatic ductal adenocarcinoma (PDAC), the third leading cause of US cancer deaths, is typically diagnosed at advanced stages,^1^ and hereditary risk factors for PDAC account for only 10% of cases.^2^ If we are to more effectively prevent PDAC and improve survival for those with PDAC,^3,4^ we must identify modifiable risk factors.

Several previous observations suggest that hepatitis C virus (HCV) may be a risk factor for PDAC. HCV is an oncovirus associated with hepatocellular carcinoma (HCC) and non-Hodgkin lymphoma^5,6,7^ that increases chronic inflammation and immune dysfunction.^7,8^ Increased HCV antigens have been found in pancreatic acinar cells. Pancreatic enzyme levels increase with worsening HCV-associated liver disease,^9^ suggesting a link between HCV and pancreatic inflammation, an established risk factor for PDAC progression.^10,11,12,13,14^

However, studies of PDAC and chronic, untreated HCV have found mixed results.^15,16,17,18,19,20,21,22,23,24^ Some studies have shown no significant association between HCV and PDAC.^15,17,21^ Others have found an association,^16,18,22,23,24,25^ but findings were often attenuated after adjusting for potential confounders.^15,17,19,21^ Other studies included a limited set of potential confounders^16,24,25^ or were not generalizable to diverse populations given their geographic restriction^23,25^ or incomplete information on race and ethnicity.^18,22^ Furthermore, HCV genotypes differentially affect the risk for HCC,^5,26,27,28,29^ but it is unknown whether distinct HCV genotypes convey differential risk of PDAC. A large cohort with accurate classification of HCV status and granular clinical data on comorbidities is crucial to discover a potential independent association of chronic, untreated HCV with PDAC burden.

The largest integrated health care system in the US, the Veterans Health Administration (VA), is ideal to address these questions. Reported prevalence of HCV is 3-fold higher in VA care than in the general US population, and VA has implemented extensive HCV screening efforts.^30,31^ Furthermore, VA has used a paperless electronic health record since 1999, allowing excellent ascertainment of HCV status and granular data on comorbidity.^32^ Our aims were (1) to measure the association of chronic untreated HCV and incident PDAC among veterans, and (2) to investigate whether distinct HCV genotypes differentially affect PDAC risk.

## Methods

### Data Source

We conducted a retrospective cohort study using data from the VA, which include longitudinal inpatient and outpatient encounters, laboratory and imaging results, and medications.^33^ This study was conducted in accordance with the Declaration of Helsinki^34^ and the Declaration of Istanbul^35^ and was approved by the West Haven VA institutional review board. Given the minimal risk of this study and the difficulty in obtaining written consent, a waiver of informed consent was obtained from the institutional review board. This observational study was reported according to Strengthening the Reporting of Observational Studies in Epidemiology (STROBE) reporting guidelines.^36^

### Study Cohorts

Patients aged 20 years or older in the VA with HCV testing and at least 1 inpatient or outpatient encounter between October 1, 2001, and September 30, 2020, were included. To simulate the risk of an individual across a wide range of time, we randomly selected 1 outpatient encounter at least 1.5 years after the initial VA encounter as the index visit,^37,38^ choosing visit dates from calendar years with at least 1 common laboratory value (albumin, alanine aminotransferase, aspartate aminotransferase, cholesterol, creatinine, hemoglobin, platelets, white blood cell count, glucose, and total bilirubin) as a proxy for regular care. We excluded individuals with prevalent PDAC or metastatic solid tumor at baseline according to Charlson Comorbidity Index (CCI)^39^ criteria for metastatic cancer. Our final cohort included patients tested for HCV.

### Exposure of Interest

We defined HCV status as the following: (1) chronic HCV (positive antibody and a positive viral load [>600 IU/mL or >600 copies/mL], positive genotype, or HCV treatment data); (2) exposed to HCV only (positive antibody only, negative viral load reflex testing independent of serologic profile, or only having an *International Classification of Diseases, Ninth Revision [ICD-9]* or *International Statistical Classification of Diseases and Related Health Problems, Tenth Revision [ICD-10]* code of HCV without associated laboratory values); and (3) those without HCV (ie, non-HCV, negative HCV antibody test). To minimize risk of misclassification, we classified patients without confirmatory HCV RNA levels as being exposed to HCV. Patients who received interferon-based HCV treatment were included in the chronic HCV group in the primary analysis.

### Outcome of Interest

The outcome of interest was the time to earliest PDAC diagnosis, defined as at least 1 inpatient or 2 outpatient encounters with PDAC *ICD-9* or *ICD-10* codes in VA or Centers for Medicare & Medicaid Services (CMS) (eTable 1 in Supplement 1). We included CMS diagnoses because a large proportion of veterans older than 65 years use dual VA-Medicare services.^40^

### Statistical Analysis

#### Primary Analyses

Data were analyzed from October 2023 to September 2025. Covariates were assessed before the index visit. Demographic variables included age (<50 years old with 5-year intervals until age 90 years), sex, and race and ethnicity (Black, Hispanic, non-Hispanic White, or other, which included American Indian or Alaska Native, Asian, Native Hawaiian or Other Pacific Islander, and multiracial) as documented from the medical record. Data on race and ethnicity were included in this study to account for social determinants of health because both HCV and PDAC incidence vary by race and ethnicity. Clinical covariates included body mass index (calculated as weight in kilograms divided by height in meters squared) categories (<18.5, 18.5 to <25.0, 25.0 to <30.0, and ≥30.0, within a range of 14-70),^41^ tobacco smoking status (current, former, or never) from the VA health factors dataset with validation from survey data^42^ and nicotine biomarkers,^43^ alcohol use disorder (AUD) defined using *ICD-9* or *ICD-10* codes,^44^ pancreatitis (acute or chronic), pancreatic cysts, and CCI as a total score (0, 1, 2, or ≥3) (eTable 1 in Supplement 1) derived from 1 inpatient or 2 outpatient encounters based on a published definition.^37^ In addition, we included individual CCI components of diabetes, HIV, and liver disease (mild or moderate to severe) as potential confounders^37,39^ (eTable 1 in Supplement 1). We grouped mild liver disease (eg, fatty liver or compensated cirrhosis) with moderate-to-severe liver disease (eg, compensated cirrhosis with complications or decompensated cirrhosis) given similar results in the analysis.

We evaluated the association of chronic HCV and incident PDAC with univariable and multivariable Cox regression models in a complete case analysis, starting from an individual’s index date, in Stata statistical software version 18 (StataCorp). To focus on chronic untreated HCV, we right-censored at the time of direct-acting antiviral (DAA) treatment, death, 2 years after the last VA or CMS encounter, or December 31, 2021, the last date of available CMS data. We calculated hazard ratios (HRs) of PDAC with 95% CIs by HCV status in unadjusted and fully adjusted models. We confirmed that the proportional hazards assumption was met by plotting log-log survival curves. We included separate interaction terms between HCV status and age, smoking, and alcohol use, as well as AUD and pancreatitis. Two-sided *P* < .10 was considered statistically significant.

In a subgroup analysis, we evaluated the association of HCV genotype with PDAC risk. HCV genotypes were categorized as genotype 1, 2, 3, or other-mixed genotype (genotypes 4-6 or a combination of any 2 genotypes). HCV genotypes 7 or higher were not observed. We calculated age-standardized incidence rate using the Fay and Feuer method,^45^ comparing against the 2020 US Census results.^46^

#### Sensitivity Analyses

We also performed several sensitivity analyses. We excluded patients who received interferon. In a separate analysis, we excluded individuals with positive HCV antibody missing HCV RNA results. We performed a sensitivity analysis eliminating individuals with any cancer at baseline (local or metastatic). Finally, to account for any potential immortal time bias, we performed an analysis including only individuals tested for HCV by their index date.

## Results

### Baseline Characteristics

Of the 6 330 856 individuals with HCV testing (Table 1), the median (IQR) age was 61.6 (49.9-70.1) years, 5 841 571 (92.3%) were men, 489 285 (7.7%) were women, 377 142 (6.0%) were Hispanic, 1 071 386 (16.9%) were non-Hispanic Black, 4 127 248 (65.2%) were non-Hispanic White, 312 657 (5.0%) were other races, and 442 423 (7.0%) were of unknown race (Table 1). There were 246 218 individuals (3.9%) with chronic HCV, 209 492 (3.3%) were exposed to HCV, and 5 875 146 (92.8%) did not have HCV infection (eFigure 1 in Supplement 1). The complete case analysis included 5 628 360 individuals with a median (IQR) follow-up time of 5.1 (2.7-9.0) years. The overall proportion of PDAC was 0.5% (33 451 individuals; 13 025 Medicare only) with a median (IQR) time to PDAC of 3.9 (1.6-7.4) years. Of those with PDAC, median (IQR) time to death was similar across HCV exposures (chronic, 0.29 [0.08-0.80] year; exposed, 0.31 [0.10-0.92] year; and without HCV, 0.32 [0.09-0.93] year). The age-standardized PDAC incidence rate was 51.92 cases per 100 000 person-years (PY) (95% CI, 51.30-52.58 cases per 100 000 PY) for non-HCV individuals, 67.97 cases per 100 000 PY (95% CI, 64.07-75.09 cases per 100 000 PY) among those exposed to HCV, and 107.69 cases per 100 000 PY (95% CI, 100.92-120.87 cases per 100 000 PY) among those with chronic HCV. There were 1520 PDAC cases (0.62%) in the group with chronic HCV, 1347 PDAC cases (0.64%) in the group exposed to HCV, and 30 148 PDAC cases (0.51%) in the group without HCV. Median age was younger among individuals with chronic HCV. In addition, there was a higher proportion of Black individuals and higher rates of smoking, alcohol use, and liver disease compared with exposed or individuals without HCV.

**Table 1.** Baseline Characteristics of HCV-Tested Veterans

Characteristic	Veterans, No. (%) (N = 6 330 856)		
	Chronic HCV (n = 246 218)	Exposed HCV (n = 209 492)	Non-HCV (n = 5 875 146)
Demographic characteristics			
Age, median (IQR), y	57.7 (52.8-62.8)	60.7 (53.1-67.9)	62.0 (49.5-70.5)
Age group, y			
<50	37 835 (15.4)	38 581 (18.5)	1 518 199 (26.0)
50-54	49 710 (20.2)	25 183 (12.1)	488 955 (8.4)
55-59	65 957 (26.8)	35 723 (17.1)	643 472 (11.0)
60-64	50 908 (20.7)	38 435 (18.4)	788 844 (13.5)
65-69	24 696 (10.0)	30 951 (14.8)	893 002 (15.3)
70-74	8862 (3.6)	18 060 (8.7)	697 537 (11.9)
75-79	4396 (1.8)	10 728 (5.1)	394 345 (6.8)
80-84	2565 (1.0)	7425 (3.6)	270 942 (4.6)
85-90	1105 (0.5)	3736 (1.8)	148 737 (2.6)
Sex			
Male	239 193 (97.1)	197 617 (94.3)	5 404 761 (92.0)
Female	7025 (2.9)	11 875 (5.7)	470 385 (8.0)
Race and ethnicity			
Black	74 811 (30.4)	45 954 (21.9)	950 621 (16.2)
Hispanic	12 324 (5.0)	12 014 (5.7)	352 804 (6.0)
White	128 484 (52.2)	126 154 (60.2)	3 872 610 (65.9)
Other^a^	15 533 (6.3)	10 753 (5.1)	286 371 (4.9)
Unknown	15 066 (6.1)	14 617 (7.0)	412 740 (7.0)
Clinical characteristics			
Body mass index^b^			
<18.5	5484 (2.2)	3918 (1.9)	58 972 (1.0)
18.5 to <25.0	79 283 (32.2)	49 267 (23.5)	1 105 207 (18.8)
25.0 to <30.0	88 690 (36.0)	71 700 (34.2)	2 100 244 (35.7)
≥30.0	67 798 (27.5)	80 090 (38.2)	2 522 179 (42.9)
Unknown	4963 (2.0)	4517 (2.2)	88 544 (1.5)
Smoking status			
Current	134 488 (54.6)	82 283 (39.3)	1 530 519 (26.1)
Former	45 743 (18.6)	51 610 (24.6)	1 559 528 (26.5)
Never	28 164 (11.4)	46 725 (22.3)	1 709 751 (29.1)
Uninterpretable	21 413 (8.7)	16 034 (7.7)	511 835 (8.7)
Unknown	16 410 (6.7)	12 840 (6.1)	563 513 (9.6)
Alcohol use disorder	115 386 (46.9)	57 211 (27.3)	874 807 (14.9)
Pancreatitis	9867 (4.0)	5799 (2.8)	92 809 (1.6)
Pancreatic cyst	807 (0.3)	703 (0.3)	14 734 (0.3)
Diabetes	57 405 (23.3)	55 192 (26.3)	1 450 918 (24.7)
HIV	7404 (3.0)	3345 (1.6)	25 387 (0.4)
Charlson Comorbidity Index score			
0	77 236 (31.4)	88 515 (42.3)	3 024 457 (51.5)
1	70 967 (28.8)	48 331 (23.1)	1 192 449 (20.3)
2	36 124 (14.7)	27 376 (13.1)	687 549 (11.7)
≥3	61 891 (25.1)	45 270 (21.6)	970 691 (16.5)
Liver disease	106 755 (43.4)	26 081 (12.4)	151 270 (2.6)
HCV genotype			
1	150 442 (61.1)	NA	NA
2	22 777 (9.3)	NA	NA
3	15 734 (6.4)	NA	NA
Other (4-6, mixed)	2416 (1.0)	NA	NA

Abbreviations: HCV, hepatitis C virus; NA, not applicable.

^a^ Other race includes American Indian or Alaska Native, Asian, Native Hawaiian or Other Pacific Islander, and multiracial.

^b^ Body mass index is calculated as weight in kilograms divided by height in meters squared.

### Incidence Rates of PDAC

**Figure 1.**
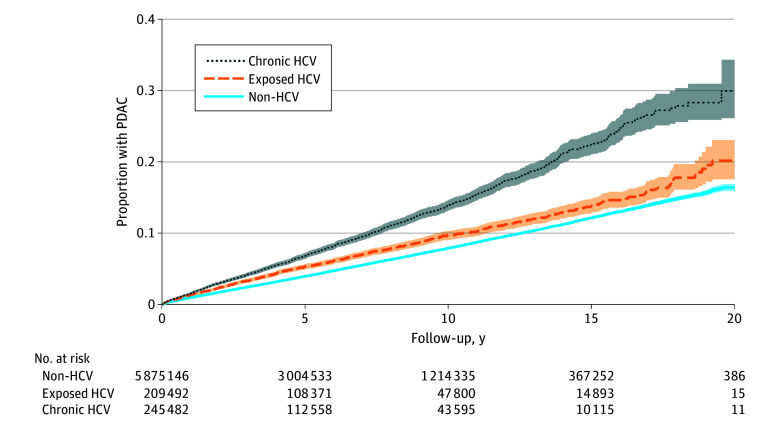
Cumulative Incidence of Pancreatic Ductal Adenocarcinoma (PDAC) by Hepatitis C Virus (HCV) Status Kaplan-Meier curve of the proportion of PDAC cases across those without HCV, those exposed to HCV, and those with chronic HCV over a 20-year period. The 95% CI is depicted as the shaded region around each line. A risk table is included below the graph at 5-year intervals.

**Figure 2.**
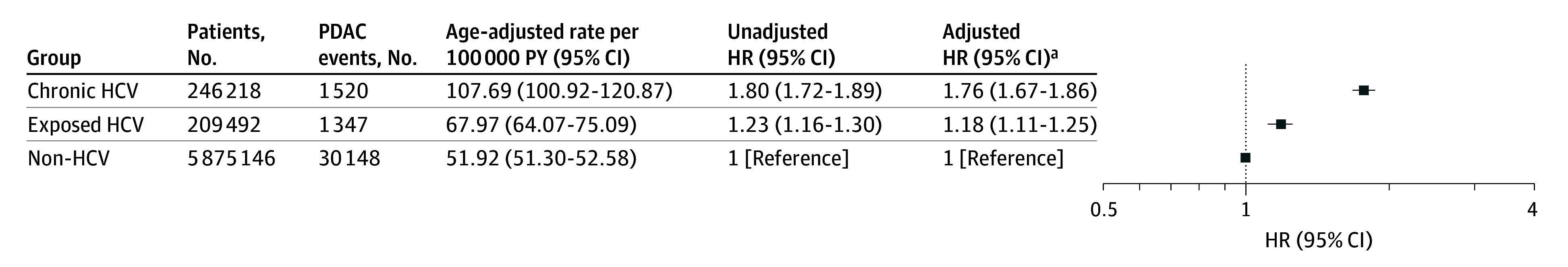
Incidence Rates and Risk of Pancreatic Ductal Adenocarcinoma (PDAC) by Hepatitis C Virus (HCV) Status Forest plot of adjusted hazard ratios (HRs) for incident PDAC by HCV status, with non-HCV as the reference group. Also shown are the total number of individuals, PDAC events, and PDAC incidence rates for each group, as well as unadjusted HRs. PY indicates person-years. ^a^Adjusted for age, sex, race, ethnicity, body mass index, smoking status, alcohol use disorder, pancreatitis, pancreatic cysts, diabetes, human immunodeficiency virus, liver disease, and Charlson Comorbidity Index score.

Individuals with chronic HCV received a PDAC diagnosis at younger ages (median [IQR] age, 65.0 [59.9-69.6] years) than those exposed to HCV (median [IQR] age, 68.5 [62.6-75.1] years) and those without HCV (median [IQR] age, 72.4 years [66.7-79.0] years) (eFigure 2 in Supplement 1). The proportion of PDAC cases was highest in those with chronic HCV, followed by those exposed to HCV and those without HCV (Figure 1). PDAC incidence rates were 143.3 cases per 100 000 PY (95% CI, 137.1-149.8 cases per 100 000 PY) for veterans with chronic HCV and 80.6 cases per 100 000 PY (95% CI, 79.7-81.5 cases per 100 000 PY) for veterans without HCV (Figure 2) and increased with each decade of age until age 80 years (Table 2). Overall, incidence rates of PDAC were higher among individuals with chronic HCV vs without HCV between 30 and 80 years of age. There was no clinically meaningful multiplicative interaction between age and HCV status (eFigure 3 in Supplement 1).

**Table 2.** Incidence Rates of PDAC Per 100 000 PY by Decade of Age in Chronic HCV and Non-HCV Cohorts

Group	Age group, y						
	20-29	30-39	40-49	50-59	60-69	70-79	80-90
Non-HCV							
Patients, No.	274 636	575 194	668 369	1 132 427	1 681 846	1 091 882	419 679
PDAC cases, No.	10	105	917	5194	11 775	8662	3342
PDAC rate, cases/100 000 PY (95% CI)	0.57 (0.31-1.06)	2.99 (2.47-3.62)	18.67 (17.50-19.92)	60.38 (58.76-62.05)	105.68 (103.79-107.61)	154.37 (151.15-157.66)	184.58 (178.43-190.95)
Exposed HCV							
Patients, No.	4561	11 818	22 202	60 906	69 386	28 788	11 161
PDAC cases, No.	0	6	72	429	477	262	96
PDAC rate, cases/100 000 PY (95% CI)	NA	6.99 (3.14-15.57)	38.76 (30.77-48.84)	94.09 (85.59-103.43)	119.09 (108-87-130.27)	185.35 (164.21-209.21)	216.00 (176.84-263.84)
IRR vs non-HCV (95% CI)	0.00 (0.00-21.71)	2.34 (0.84-5.26)	2.08 (1.61-2.64)	1.56 (1.41-1.72)	1.13 (1.03-1.23)	1.20 (1.06-1.36)	1.17 (0.95-1.43)
Chronic HCV							
Patients, No.	2726	6067	29 042	115 667	75 604	13 258	3670
PDAC cases, No.	1	5	158	962	653	146	31
PDAC rate, cases/100 000 PY (95% CI)	5.98 (0.84-42.42)	13.28 (5.53-31.90)	66.69 (57.06-77.94)	137.37 (128.96-146.33)	213.61 (197.84-230.64)	272.27 (231.50-320.22)	234.92 (165.21-334.05)
IRR vs non-HCV (95% CI)	10.50 (0.24-73.79)	4.44 (1.41-10.69)	3.57 (3.00-4.23)	2.28 (2.12-2.44)	2.02 (1.87-2.19)	1.76 (1.49-2.08)	1.27 (0.86-1.81)

Abbreviations: HCV, hepatitis C virus; IRR, incidence rate ratio; NA, not applicable; PDAC, pancreatic ductal adenocarcinoma; PY, person-years.

### Chronic HCV and PDAC Risk

Compared with persons who did not have HCV, individuals with HCV exposure but no active infection at baseline (unadjusted HR, 1.23; 95% CI, 1.16-1.30) and those with chronic HCV (unadjusted HR, 1.80; 95% CI, 1.72-1.89) had higher risks of PDAC (Figure 2). This association persisted after adjusting for covariates (HCV exposed, adjusted HR [aHR], 1.18; 95% CI, 1.11-1.25; chronic HCV, aHR, 1.76; 95% CI, 1.67-1.86) (Figure 2). Covariates significantly associated with incident PDAC by decreasing magnitude were age, pancreatic cysts, pancreatitis, male sex, current smoking, CCI score, diabetes, body mass index less than 18.5, HIV, liver disease, AUD, and former smoking (eTable 2 in Supplement 1). There were no significant interactions found for smoking, AUD, and pancreatitis.

### HCV Genotype and PDAC Risk

Over three-quarters of individuals (191 369 individuals [77.7%]) with chronic HCV had genotype data (150 442 individuals [61.1%] with genotype 1, 22 777 individuals [9.3%] with genotype 2, and 15 734 individuals [6.4%] with genotype 3). All HCV genotypes were associated with increased risk for PDAC (Table 3) with hazards in decreasing order: other or mixed genotype (aHR, 2.18; 95% CI, 1.43-3.31), genotype 3 (aHR, 2.02; 95% CI, 1.67-2.45), genotype 1 (aHR, 1.75; 95% CI, 1.64-1.87), and genotype 2 (aHR, 1.35; 95% CI, 1.14-1.60).

**Table 3.** HCV Genotype Associations

Group	Patients, No.	PDAC events, No.	Adjusted HR (95% CI)^a^
Non-HCV	5 211 406	26 317	1 [Reference]
Exposed HCV	192 214	1226	1.18 (1.11-1.25)
Chronic HCV			
Genotype 1	138 901	1104	1.75 (1.64-1.87)
Genotype 2	20 966	141	1.35 (1.14-1.60)
Genotype 3	14 586	106	2.02 (1.67-2.45)
Other genotype^b^	2242	22	2.18 (1.43-3.31)

Abbreviations: HCV, hepatitis C virus; HR, hazard ratio; PDAC, pancreatic ductal adenocarcinoma.

^a^ Adjusted for age, sex, race, ethnicity, body mass index, smoking status, alcohol use disorder, pancreatitis, pancreatic cysts, diabetes, human immunodeficiency virus, liver disease, and Charlson Comorbidity Index score.

^b^ Other HCV genotypes include genotypes 4 to 6 and mixed genotypes.

### Sensitivity Analyses

Overall, 32 627 individuals (16.1%) with chronic HCV received interferon-based treatment. In addition, 76 416 individuals (1.2%) in the entire cohort with a positive HCV antibody had no reported HCV RNA. Our 2 sensitivity analyses excluding these individuals showed similar results to the primary analysis (eTable 2 in Supplement 1). We performed additional sensitivity analyses, excluding individuals with any prior cancer at baseline (eTable 3 in Supplement 1) or including only patients who received HCV testing up to the index date (eTable 4 in Supplement 1), again with results similar to those of the main analysis.

## Discussion

In this cohort study of more than 6 million veterans tested for HCV, compared with no HCV infection, chronic HCV infection was associated with an increased risk of PDAC. This remained true after adjusting for risk factors, including tobacco smoking, alcohol use, diabetes, pancreatitis, and liver disease. Among those with chronic HCV, we found that HCV genotypes 1 and 3 were associated with higher risk of PDAC compared with genotype 2. We also found that individuals with chronic HCV received a diagnosis of PDAC at younger ages than those exposed to HCV and those without HCV. In addition, individuals exposed to HCV had higher risk of PDAC compared with individuals without HCV.

A major strength of this study is its large, national sample with a substantial HCV prevalence. We used a previously studied HCV phenotype algorithm incorporating laboratory and treatment data to distinguish chronic untreated HCV from previously exposed or treated cases.^37^ Individuals with positive HCV antibodies but unknown viral load were conservatively categorized in the HCV exposed group, and excluding these individuals did not meaningfully alter our findings. The fact that we linked VA and CMS data to identify a greater number of PDAC cases is a major strength. The granular clinical data available through the VA and CMS also permitted us to adjust for relevant confounders, including alcohol use and tobacco smoking, which are significant risk factors for pancreatic cancer.^2^

Although other studies have investigated the association between HCV and PDAC risk, these have been limited by sample size,^15,17,21^ geographic generalizability,^15,17,21,22,23,25,47^ or the availability of potential confounding risk factors^16,17,24,25^ (eTable 5 in Supplement 1). Specifically, several prior studies similarly found an increased adjusted risk of PDAC among patients with HCV.^16,18,22,23,25^ In a previous large study of veterans, El-Serag et al^19^ demonstrated a higher risk of PDAC in veterans with HCV, but the association was attenuated after adjusting for alcohol use, pancreatitis, cholelithiasis, and primary sclerosing cholangitis. That study^48^ only considered PDAC cases diagnosed within the VA, missing a substantial number of cases documented in Medicare data only. Because they relied on *ICD-9* or *ICD-10* codes to classify HCV, they were unable to distinguish those with chronic HCV infection from those who were only exposed. Other studies finding no association between HCV and PDAC considered only seropositivity, rather than requiring confirmatory laboratory values or *ICD-9* or *ICD-10* codes.^15,17,21^ As our results suggest, this approach likely attenuated the association between HCV infection and PDAC.

Our findings suggest an independent association between chronic HCV infection and PDAC. Although future studies are needed to determine whether HCV treatment with DAA therapy partially or completely mitigates the observed PDAC risk, it is important to emphasize that untreated HCV is modifiable. Most identified clinical risk factors associated with PDAC are either nonmodifiable (eg, age) or difficult to treat (eg, smoking or obesity). In contrast, treatment with DAA therapy has a cure rate exceeding 95%.^49,50^ The VA has treated more than 80% of HCV-infected veterans since the implementation of DAA treatment in 2014.^51^ In non-VA health settings both in the US and globally, costs and access to HCV testing and treatment are major barriers.^52,53^ Our study lends additional support for HCV treatment initiatives.

Future pancreatic cancer early detection prediction models should consider whether inclusion of untreated HCV or HCV exposure further improves identification of individuals at risk. Importantly, future work should address whether HCV treatment with DAA mitigates the risk of PDAC associated with HCV infection. Given the potential selection bias among individuals chosen for DAA therapy (eg, practitioner selection bias or access to care bias), future studies will need to address potential confounding through a different analytical approach, such as propensity score weighting, which is outside the scope of this project.

Our study also suggests that distinct HCV genotypes are associated with varying risks of PDAC. Although HCV genotypes have not been studied in relation to PDAC, earlier studies of HCC found either genotype 3^26^ or genotype 1^5,27,28,29^ were associated with higher risk of HCC. It is unknown whether this association is due to inherent differences in the pathogenicity of HCV genotypes or unmeasured confounding.

Although the mechanisms of the association between HCV and PDAC remain unclear, it may be related to chronic inflammatory changes and/or viral replication in the pancreas.^54,55,56^ Chronic HCV may create an inflammatory microenvironment for neovascularization and tumor growth in the pancreas, as has been seen in other cancers, such as HCC.^57^ HCV-induced liver fibrosis is mediated, in part, by activation of hepatic stellate cells, a major component of the HCC tumor microenvironment.^58,59^ Pancreatic stellate cells are similarly involved in fibrosis of the pancreas and contribute to the severe desmoplastic tumor microenvironment in PDAC.^60^ Known PDAC inflammatory risk factors, including alcohol use, cigarette smoking, and diabetes, contribute to pancreatic stellate cells activation,^61^ but the association of HCV and pancreatic stellate cell activation has not yet been investigated. In addition, HCV found in acinar cells may lead to direct genetic alteration.^13^ Further translational investigations of these potential mechanisms as they relate to PDAC progression are needed to better understand the pathogenicity of HCV.

### Limitations

Our study has limitations. First, we focused on veterans in care, a predominantly male population, which could limit the generalizability of our findings to women. However, the incidence of PDAC is similar across sexes.^1^ Second, toxic military exposures, such as Agent Orange, can increase the risk of cancer,^62^ so future studies should account for military exposures as data become available.^63^ However, the risk has been highest for hematologic cancers, not solid tumors.^64^ In addition, the association between HCV and PDAC could be influenced by access to health care and socioeconomic status, which are not fully accounted for in our study. The effect of these factors may be mitigated in VA as it is a relatively equal-access health care system. We did not a priori exclude patients who had received interferon-based treatment, since these treatments have a low (40%-50%) probability of sustained viral response with many adverse effects^65^ and, thus, were not widely implemented in the VA. Our sensitivity analysis excluding interferon-treated patients revealed results similar to those of our primary analysis. Thus, their inclusion did not significantly influence the association between chronic HCV and incident PDAC. Our criteria of excluding metastatic cancer at baseline was based on the published CCI criteria for metastatic cancer, which may introduce some misclassification. There could also be surveillance bias if individuals with chronic HCV underwent more routine imaging that could identify more incidental PDAC. Although we were unable to identify stage of PDAC diagnosis, the median time to death was similar among exposure groups. Furthermore, because CMS data are claims based, we were only able to include individuals in our study who had HCV testing or treatment performed at the VA only. However, our sample of more than 6 million individuals had adequate power to detect the positive association.

## Conclusions

In this cohort study, we found that chronic HCV infection is associated with incident PDAC. Among those with HCV, genotype 1 (most common genotype) and 3 had elevated PDAC risk. Future work should focus on the impact of DAA treatment for HCV on risk mitigation of PDAC and mechanisms of PDAC carcinogenesis related to chronic HCV infection.

## Notes

Supplement 1. eTable 1. Covariate *ICD* codes eTable 2. PDAC Risk Excluding Interferon-treated Patients or those with Positive Antibody/Unknown RNA Levels eTable 3. PDAC Risk Excluding those with Localized Solid Tumor eTable 4. PDAC Risk Among Individuals with HCV Testing Prior to Index Date eTable 5. Summary of studies on the association of HCV and pancreatic cancer eFigure 1. Study Cohort Flowchart eFigure 2. Age at Pancreatic Cancer Diagnosis by HCV Status eFigure 3. Hazards for PDAC across Age among individuals with Chronic HCV eReferences

Supplement 2. Data Sharing Statement
