# Supplementary material for: Pancreatic Ductal Adenocarcinoma After Hepatitis C Infection
Source: JAMA Netw Open. 2025 Nov 14;8(11):e2543701. doi: 10.1001/jamanetworkopen.2025.43701 (PMC12619103; doi:10.1001/jamanetworkopen.2025.43701)
Supplement: Supplement 1. — eTable 1. Covariate ICD codes eTable 2. PDAC Risk Excluding Interferon-treated Patients or those with Positive Antibody/Unknown RNA Levels eTable 3. PDAC Risk Excluding those with Localized Solid Tumor eTable 4. PDAC Risk Among Individuals with HCV Testing Prior to Index Date eTable 5. Summary of studies on the association of HCV and pancreatic cancer eFigure 1. Study Cohort Flowchart eFigure 2. Age at Pancreatic Cancer Diagnosis by HCV Status eFigure 3. Hazards for PDAC across Age among individuals with Chronic HCV eReferences [file jamanetwopen-e2543701-s001.pdf]

Supplemental Online Content

Levinson RN, Bushman R, Tate JP, et al. Pancreatic ductal adenocarcinoma after hepatitis C infection. *JAMA Netw Open*. 2025;8(11):e2543701. doi:10.1001/jamanetworkopen.2025.43701

eTable 1. Covariate *ICD* codes
eTable 2. PDAC Risk Excluding Interferon-treated Patients or those with Positive Antibody/Unknown RNA Levels
eTable 3. PDAC Risk Excluding those with Localized Solid Tumor
eTable 4. PDAC Risk Among Individuals with HCV Testing Prior to Index Date
eTable 5. Summary of studies on the association of HCV and pancreatic cancer
eFigure 1. Study Cohort Flowchart
eFigure 2. Age at Pancreatic Cancer Diagnosis by HCV Status
eFigure 3. Hazards for PDAC across Age among individuals with Chronic HCV
eReferences

This supplemental material has been provided by the authors to give readers additional information about their work.

© 2025 Levinson RN et al. *JAMA Network Open*.

eTable 1. **Covariate *ICD* codes**

	**ICD-10 Codes**	**ICD-9 Codes**
AUD	F10.1x, F10.2x	303.0x, 303.9x, 305.0x
Diabetes	Without complications: E10.0, E10.1, E10.6, E10.8, E10.9, E11.0, E11.1, E11.6, E11.8, E11.9, E13.0, E13.1, E13.6, E13.8, E13.9<br>With complications: E10.2-E10.5, E11.2-E11.5, E13.2-E13.5	Without complications: 250.0-250.3, 250.8, 250.9<br>With complications: 250.4-250.7
Liver disease	Mild: B18.x, K73.x, K74.x, K70.0-K70.3, K70.9, K71.3-K71.5, K71.7, K75.4, K76.0, K76.2-K76.4, K76.8, K76.9, Z94.4<br>Moderate/severe: I85.0, I85.9, I86.4, I98.2, K70.4, K71.1, K72.1, K72.9, K76.5-K76.7	Mild: 070.22, 070.23, 070.32, 070.33, 070.44, 070.54, 070.6, 070.9, 570.x, 571.x, 573.3, 573.4, 573.8, 573.9, V42.7<br>Moderate/severe: 456.0-456.2, 572.2-572.8
HCV	B18.2	070.44, 070.54
HIV	B20.x-B22.x, B24	042-044
Pancreatic cyst	K86.2, K86.3	577.2
Pancreatitis	Acute: K85.x<br>Chronic: K86.0, K86.1	Acute: 577.0<br>Chronic: 577.1
PDAC	C25.0-C25.3, C25.7-C25.9	157.0-157.3, 157.7-157.9

© 2025 Levinson RN et al. *JAMA Network Open*.

eTable 2. **PDAC Risk Excluding Interferon-treated Patients or those with Positive Antibody/Unknown RNA Levels**

	Adjusted Hazard Ratio (95% CI)		
**Characteristic**	**Primary Model (n= 5,628,360)**	**Excluding Interferon-treated Patients (n= 5,592,057)**	**Excluding Patients with Positive Antibody + Unknown RNA Levels (n=5,557,701)**
HCV			
Non-HCV	1 [Reference]	1 [Reference]	1 [Reference]
Exposed HCV	1.18 (1.11-1.25)	1.18 (1.11-1.25)	1.10 (1.02-1.19)
Chronic HCV	1.76 (1.67-1.86)	1.81 (1.71-1.92)	1.76 (1.67-1.86)
Age			
<50	0.28 (0.26-0.30)	0.28 (0.26-0.30)	0.28 (0.26-0.30)
50-54	1 [Reference]	1 [Reference]	1 [Reference]
55-59	1.49 (1.41-1.57)	1.48 (1.40-1.56)	1.50 (1.42-1.58)
60-64	1.99 (1.89-2.09)	1.98 (1.88-2.09)	2.01 (1.90-2.11)
65-69	2.47 (2.34-2.60)	2.46 (2.33-2.59)	2.49 (2.36-2.63)
70-74	2.94 (2.78-3.11)	2.93 (2.77-3.09)	2.97 (2.81-3.14)
75-79	3.57 (3.37-3.79)	3.55 (3.34-3.77)	3.60 (3.39-3.82)
80-84	3.79 (3.54-4.05)	3.76 (3.52-4.02)	3.83 (3.58-4.10)
85-90	3.68 (3.38-4.01)	3.65 (3.35-3.98)	3.73 (3.42-4.06)
Sex			
Female	0.69 (0.64-0.74)	0.69 (0.64-0.74)	0.69 (0.64-0.74)

© 2025 Levinson RN et al. *JAMA Network Open*.

Male	1 [Reference]	1 [Reference]	1 [Reference]
Race/Ethnicity			
Black	1.13 (1.10-1.17)	1.13 (1.09-1.17)	1.13 (1.10-1.17)
Hispanic	0.88 (0.83-0.93)	0.88 (0.83-0.93)	0.88 (0.83-0.93)
White	1 [Reference]	1 [Reference]	1 [Reference]
Other	0.99 (0.94-1.05)	0.99 (0.94-1.05)	0.99 (0.94-1.05)
Unknown	1.57 (1.51-1.63)	1.57 (1.51-1.63)	1.57 (1.51-1.63)
BMI			
Underweight (<18.5)	1.24 (1.12-1.38)	1.24 (1.12-1.38)	1.23 (1.11-1.37)
Healthy weight (18.5-25)	1 [Reference]	1 [Reference]	1 [Reference]
Overweight (25-30)	0.91 (0.88-0.94)	0.91 (0.88-0.94)	0.91 (0.88-0.94)
Obese (>30)	0.93 (0.90-0.96)	0.93 (0.90-0.96)	0.94 (0.91-0.97)
Smoking Status			
Current	1.44 (1.40-1.49)	1.44 (1.39-1.49)	1.44 (1.40-1.49)
Former	1.04 (1.01-1.07)	1.04 (1.01-1.07)	1.04 (1.01-1.08)
Never	1 [Reference]	1 [Reference]	1 [Reference]
Uninterpretable	1.18 (1.13-1.22)	1.18 (1.13-1.22)	1.18 (1.13-1.22)
Charlson Comorbidity Index			
CCI 0	1 [Reference]	1 [Reference]	1 [Reference]
CCI 1	1.18 (1.14-1.22)	1.18 (1.14-1.22)	1.18 (1.14-1.22)
CCI 2	1.29 (1.24-1.34)	1.29 (1.24-1.34)	1.29 (1.24-1.34)
CCI ≥3	1.35 (1.29-1.40)	1.35 (1.39-1.40)	1.35 (1.30-1.41)
Liver disease	1.16 (1.10-1.22)	1.17 (1.10-1.23)	1.17 (1.10-1.23)
AUD	1.13 (1.09-1.17)	1.13 (1.09-1.16)	1.13 (1.09-1.16)
Pancreatitis	2.20 (2.08-2.33)	2.22 (2.09-2.35)	2.19 (2.07-2.33)
Pancreatic cyst	3.60 (3.25-3.98)	3.58 (3.24-3.96)	3.63 (3.28-4.01)

© 2025 Levinson RN et al. *JAMA Network Open*.

Diabetes	1.29 (1.25-1.33)	1.29 (1.25-1.32)	1.29 (1.25-1.32)
HIV	1.20 (1.03-1.38)	1.17 (1.01-1.36)	1.20 (1.03-1.39)

AUD = alcohol use disorder; BMI = body mass index; CCI = Charlson comorbidity index; CI = confidence interval; HCV = hepatitis C virus; HIV = human immunodeficiency virus; PDAC = pancreatic ductal adenocarcinoma

© 2025 Levinson RN et al. *JAMA Network Open*.

eTable 3. **PDAC Risk Excluding those with Localized Solid Tumor**

	**Adjusted Hazard Ratio (95% CI)**
**Characteristic**	**Excluding those with Localized Solid Tumor (n= 5,169,316)**
HCV	
Non-HCV	1 [Reference]
Exposed HCV	1.20 (1.13-1.27)
Chronic HCV	1.78 (1.68-1.88)
Age	
<50	0.28 (0.26-0.30)
50-54	1 [Reference]
55-59	1.51 (1.43-1.60)
60-64	2.03 (1.93-2.14)
65-69	2.54 (2.40-2.68)
70-74	3.07 (2.90-3.26)
75-79	3.74 (3.51-3.98)
80-84	3.97 (3.69-4.26)
85-90	3.80 (3.45-4.19)
Sex	
Female	0.69 (0.63-0.74)
Male	1 [Reference]
Race/Ethnicity	
Black	1.13 (1.10-1.18)
Hispanic	0.84 (0.79-0.90)
White	1 [Reference]

© 2025 Levinson RN et al. *JAMA Network Open*.

Other	0.99 (0.93-1.04)
Unknown	1.54 (1.47-1.60)
BMI	
Underweight (<18.5)	1.29 (1.15-1.44)
Healthy weight (18.5-25)	1 [Reference]
Overweight (25-30)	0.91 (0.88-0.94)
Obese (>30)	0.94 (0.91-0.98)
Smoking Status	
Current	1.48 (1.43-1.53)
Former	1.05 (1.02-1.09)
Never	1 [Reference]
Uninterpretable	1.17 (1.12-1.22)
Charlson Comorbidity Index	
CCI 0	1 [Reference]
CCI 1	1.15 (1.11-1.19)
CCI 2	1.20 (1.14-1.25)
CCI ≥3	1.18 (1.13-1.24)
Liver disease	1.18 (1.11-1.26)
AUD	1.14 (1.11-1.19)
Pancreatitis	2.26 (2.12-2.41)
Pancreatic cyst	3.49 (3.12-3.91)
Diabetes	1.34 (1.29-1.38)
HIV	1.31 (1.12-1.53)

AUD = alcohol use disorder; BMI = body mass index; CCI = Charlson comorbidity index; CI = confidence interval; HCV = hepatitis C virus; HIV =

© 2025 Levinson RN et al. *JAMA Network Open*.

human immunodeficiency virus; PDAC = pancreatic ductal adenocarcinoma

© 2025 Levinson RN et al. *JAMA Network Open*.

eTable 4. **PDAC Risk Among Individuals with HCV Testing Prior to Index Date**

	**Adjusted Hazard Ratio (95% CI)**
**Characteristic**	**Excluding Individuals Tested for HCV after Index Date (n= 4,401,904)**
HCV	
Non-HCV	1 [Reference]
Exposed HCV	1.15 (1.07-1.23)
Chronic HCV	1.75 (1.65-1.86)
Age	
<50	0.28 (0.26-0.31)
50-54	1 [Reference]
55-59	1.54 (1.45-1.64)
60-64	2.04 (1.92-2.17)
65-69	2.49 (2.34-2.64)
70-74	2.90 (2.72-3.09)
75-79	3.43 (3.20-3.67)
80-84	3.52 (3.26-3.81)
85-90	3.29 (2.98-3.63)
Sex	
Female	0.70 (0.64-0.76)
Male	1 [Reference]
Race/Ethnicity	
Black	1.11 (1.07-1.15)
Hispanic	0.87 (0.82-0.93)

© 2025 Levinson RN et al. *JAMA Network Open*.

White	1 [Reference]
Other	0.99 (0.93-1.05)
Unknown	1.57 (1.50-1.64)
BMI	
Underweight (<18.5)	1.20 (1.07-1.34)
Healthy weight (18.5-25)	1 [Reference]
Overweight (25-30)	0.89 (0.86-0.93)
Obese (>30)	0.91 (0.88-0.95)
Smoking Status	
Current	1.40 (1.35-1.45)
Former	1.03 (0.99-1.06)
Never	1 [Reference]
Uninterpretable	1.19 (1.14-1.24)
Charlson Comorbidity Index	
CCI 0	1 [Reference]
CCI 1	1.17 (1.12-1.21)
CCI 2	1.26 (1.21-1.32)
CCI ≥3	1.32 (1.26-1.38)
Liver disease	1.14 (1.08-1.21)
AUD	1.10 (1.06-1.14)
Pancreatitis	2.20 (2.07-2.35)
Pancreatic cyst	3.57 (3.20-3.98)
Diabetes	1.31 (1.27-1.36)
HIV	1.23 (1.06-1.43)

AUD = alcohol use disorder; BMI = body mass index; CCI = Charlson comorbidity index; CI = confidence interval; HCV = hepatitis C virus; HIV = human immunodeficiency virus; PDAC = pancreatic ductal adenocarcinoma

© 2025 Levinson RN et al. *JAMA Network Open*.

eTable 5. **Summary of studies on the association of HCV and pancreatic cancer**

Study (author, year)	Cohort	Study design	Study period	Sample size	HCV definition	Covariates adjusted for	Effect estimates (95% CI)
**Studies with Significant Associations**							
Allison et al., 2015 [1]	Chronic hepatitis cohort study (CHeCS)[1] compared to SEER	Retrospective cohort study	2006-2010	2,143,369 subjects	ICD-9 code and/or laboratory data (positive HCV antibody, RNA, or genotype)	Age	SRR: 2.5 (1.7-3.2)
Darvishian et al., 2021 [2]	British Columbia Hepatitis Testers Cohort	Retrospective cohort study	1990-2015	658,697 subjects	Positive HCV antibody, HCV RNA, or genotype	Age, sex, ethnicity, problematic alcohol use, injection drug use, cirrhosis, diabetes, material and social deprivation, Elixhauser comorbidity index	aHR 2.79 (2.01-3.70)
El-Serag et al., 2009 [3]	Veterans Affairs	Retrospective cohort study	1998-2004	718,687 subjects	ICD-9 code on 2 visits	Matching variables: age, sex, baseline visit date, type of visit	aHR (matching variables only): 1.23 (1.02-1.49)<br>Additionally adjusted for Acute pancreatitis: 1.18 (0.97-1.42)<br>Chronic pancreatitis: 1.18 (0.98-1.42)<br>Alcoholism: 1.21 (1.00-1.46)<br>Alcoholic liver disease: 1.18 (0.98-1.44)<br>Cholecystectomy: 1.23 (1.02-1.49)

© 2025 Levinson RN et al. *JAMA Network Open*.

							Choledochal cyst: 1.23 (1.02-1.48)<br>Choledocholithiasis: 1.21 (1.00-1.46)<br>Cholelithiasis: 1.18 (0.97-1.42)<br>Cirrhosis: 1.12 (0.92-1.37)<br>Diabetes: 1.24 (1.03-1.49)<br>HBV: 1.19 (0.98-1.44)<br>IBD: 1.23 (1.02-1.49)<br>PSC: 1.20 (0.99-1.45)
Huang et al., 2013 [4]	Swedish Institute for Infectious Disease Control and Population Register at Statistics Sweden	Retrospective matched cohort study	1990-2008	236,650 subjects	Positive HCV antibody or HCV RNA	Age, sex, county, COPD, diabetes, chronic pancreatitis, alcohol use disorder-related diagnoses	aHR: 1.6 (1.04-2.4)
Lam et al., 2021 [5]	Kaiser Permanente Northern California (KPNC)	Retrospective cohort study	2007-2017	175,999 subjects	Positive HCV RNA or genotype	Age, sex, race/ethnicity, calendar year, smoking status, alcohol/drug use disorder, HBV, HIV	aIRR: 2.0 (1.6-2.5)
Woo et al., 2013 [6]	National Cancer Center in Korea	Case-control study	2001-2011	3,765 subjects	Positive HCV antibody	Age, gender, smoking status, diabetes	aOR: 2.30 (1.30-4.08)

© 2025 Levinson RN et al. *JAMA Network Open*.

**Studies with Non-Significant Associations**							
Abe et al., 2016 [7]	Japan Public Health Center (JPHC)	Prospective study	1993-2010	20,360 subjects	Positive HCV antibody	Sex, age, study area, BMI, diabetes, smoking status	aHR: 0.69 (0.28-1.69)
Chang et al., 2014 [8]	National Taiwan University Hospital	Case-control study	2000-2013	2,301 subjects	Positive HCV antibody	Age, sex, diabetes, smoking	aOR: 1.36 (0.80-2.31)
Hassan et al., 2008 [9]	University of Texas M.D. Anderson Cancer Center	Case-control study	2000-2007	1,355 subjects	Positive HCV antibody	Age, sex, race, state of residency, educational level, smoking, diabetes, alcohol, family history of cancer	aOR: 0.9 (0.3-2.8)

[1] Among four US healthcare systems: Geisinger Health System (PA), Henry Ford Health System (MI), Kaiser Permanente Northwest (OR), Kaiser Permanente Hawaii (HI)
aHR = adjusted HR; aIRR = adjusted incidence rate ratio; aOR = adjusted OR; BMI = body mass index; CI = confidence interval; COPD = chronic obstructive pulmonary disease; HBV = hepatitis B virus; HCV = hepatitis C virus; HIV = human immunodeficiency virus; IBD = inflammatory bowel disease; ICD = International Classification of Diseases; PSC = primary sclerosing cholangitis; RNA = ribonucleic acid; SEER = Surveillance, Epidemiology, and End Results Program; SRR = standardized rate ratio

© 2025 Levinson RN et al. *JAMA Network Open*.

eFigure 1. **Study Cohort Flowchart**

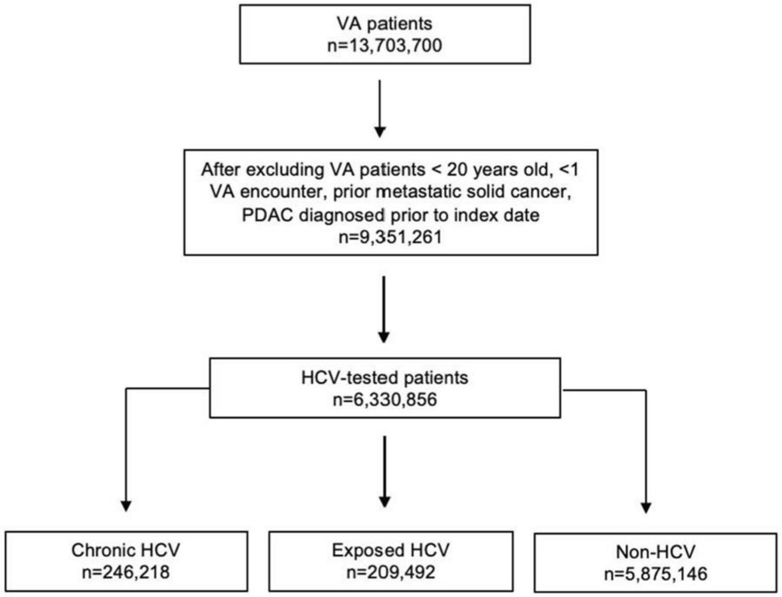

eFigure 1: Study Cohort Flowchart
VA patients from 2001-2021 were considered for this study. Exclusion criteria were based on age, service utilization, cancer status, and HCV testing. The final cohort was divided into three groups based on HCV status.
HCV = hepatitis C virus; PDAC = pancreatic ductal adenocarcinoma; VA = Veterans Health Administration

© 2025 Levinson RN et al. *JAMA Network Open*.

eFigure 2. **Age at Pancreatic Cancer Diagnosis by HCV Status**

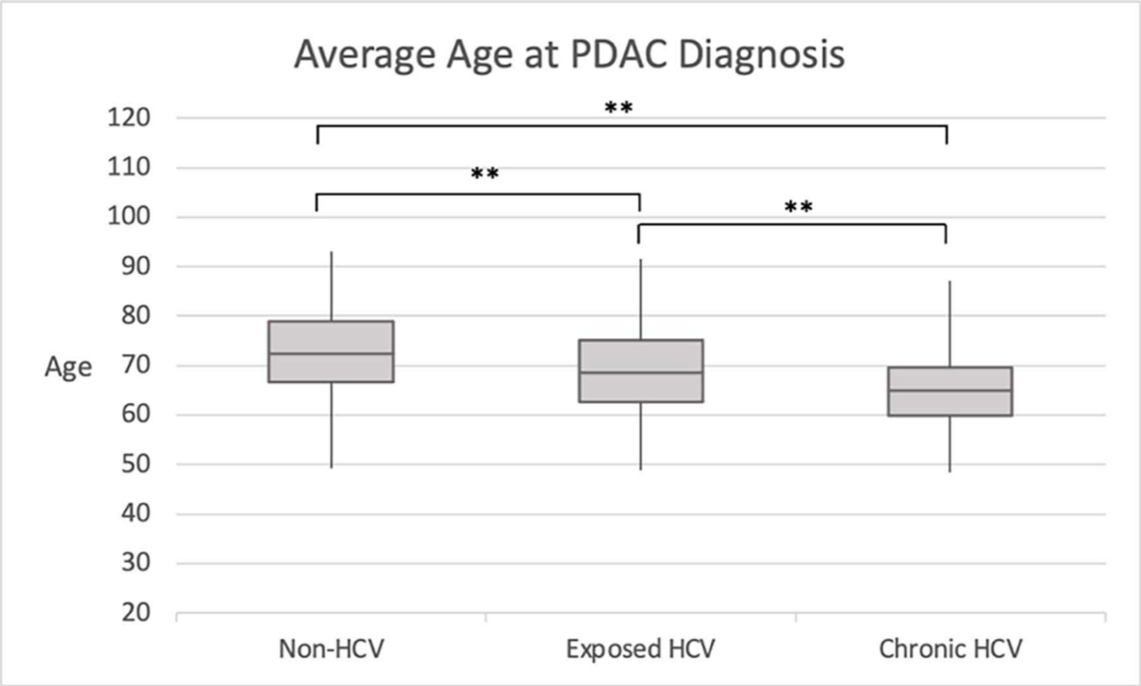

eFigure 2: Age at Pancreatic Cancer Diagnosis by HCV Status
Box plot of age at PDAC diagnosis across HCV status: chronic HCV (median: 65.0 years, IQR:59.9-69.6), HCV exposed (median: 68.5 years, IQR: 62.6-75.1), non-HCV (median: 72.4 years, IQR: 66.7-79.0)
** = p<0.01
HCV = hepatitis C virus; PDAC = pancreatic ductal adenocarcinoma

© 2025 Levinson RN et al. *JAMA Network Open*.

eFigure 3. **Hazards for PDAC across Age by HCV Status**

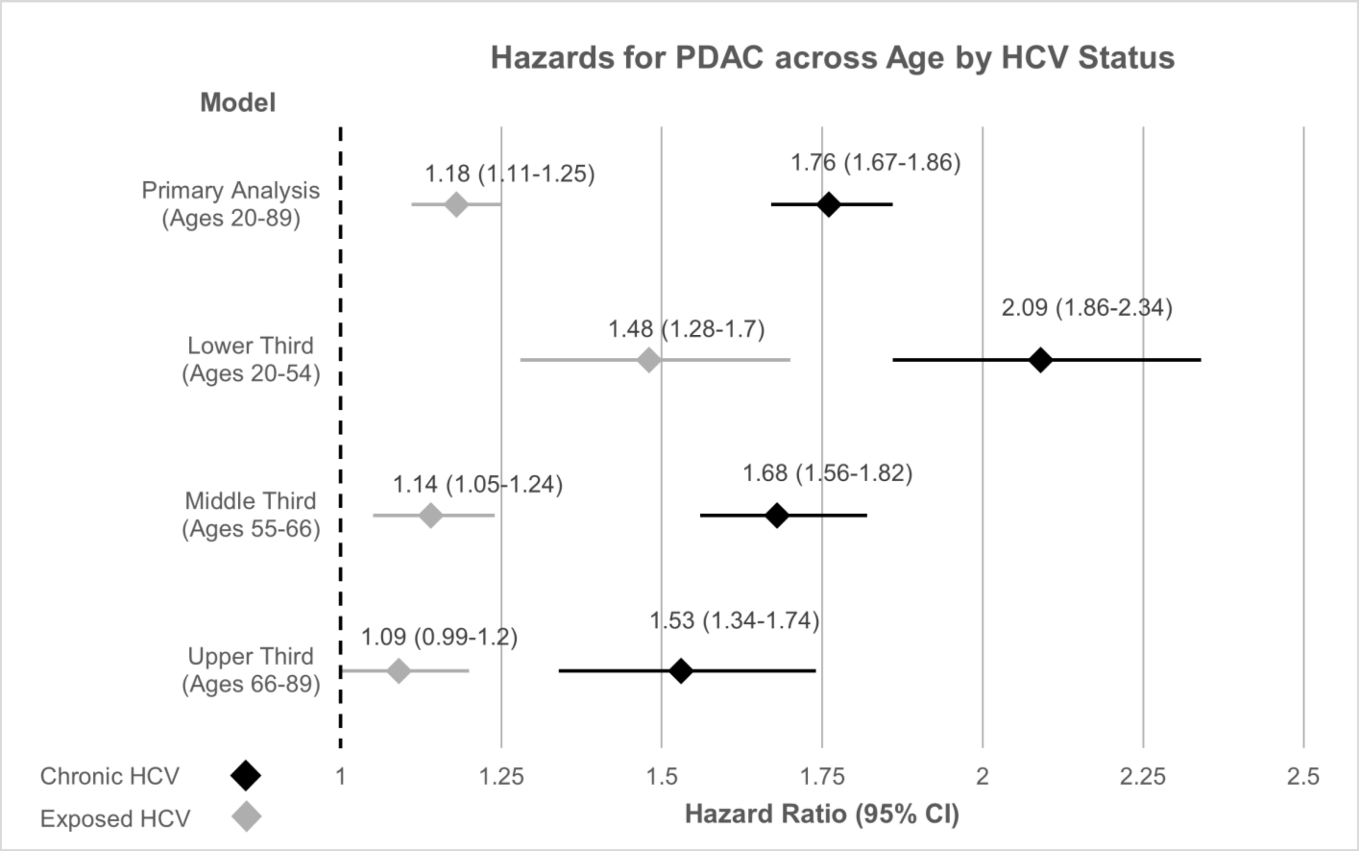

eFigure 3: Hazards for PDAC across Age by HCV Status
Forest plot of HCV status adjusted hazard ratios and 95% confidence intervals for PDAC diagnosis, evaluating all ages and age separated in tertiles.
HCV = hepatitis C virus; PDAC = pancreatic ductal adenocarcinoma

© 2025 Levinson RN et al. *JAMA Network Open*.

eReferences

1. Allison RD, Tong X, Moorman AC, et al. Incidence of Cancer and Cancer-related Mortality Among Persons with Chronic Hepatitis C Infection, 2006–2010. *J Hepatol*. 2015;63(4):822-828. doi:10.1016/j.jhep.2015.04.021

2. Darvishian M, Butt ZA, Wong S, et al. Elevated risk of colorectal, liver, and pancreatic cancers among HCV, HBV and/or HIV (co)infected individuals in a population based cohort in Canada. *Ther Adv Med Oncol*. 2021;13:1758835921992987. doi:10.1177/1758835921992987

3. El-Serag HB, Engels EA, Landgren O, et al. Risk of hepatobiliary and pancreatic cancers after hepatitis C virus infection: A population-based study of U.S. veterans. *Hepatology*. 2009;49(1):116-123. doi:10.1002/hep.22606

4. Huang J, Magnusson M, Törner A, Ye W, Duberg AS. Risk of pancreatic cancer among individuals with hepatitis C or hepatitis B virus infection: a nationwide study in Sweden. *Br J Cancer*. 2013;109(11):2917-2923. doi:10.1038/bjc.2013.689

5. Lam JO, Hurley LB, Lai JB, et al. Cancer in people with and without hepatitis C virus infection: comparison of risk before and after introduction of direct-acting antivirals. *Cancer Epidemiol Biomarkers Prev*. 2021;30(12):2188-2196. doi:10.1158/1055-9965.EPI-21-0742

6. Woo SM, Joo J, Lee WJ, et al. Risk of Pancreatic Cancer in Relation to ABO Blood Group and Hepatitis C Virus Infection in Korea: A Case-Control Study. *J Korean Med Sci*. 2013;28(2):247-251. doi:10.3346/jkms.2013.28.2.247

7. Abe SK, Inoue M, Sawada N, et al. Hepatitis B and C Virus Infection and Risk of Pancreatic Cancer: A Population-Based Cohort Study (JPHC Study Cohort II). *Cancer Epidemiology, Biomarkers & Prevention*. 2016;25(3):555-557. doi:10.1158/1055-9965.EPI-15-1115

8. Chang MC, Chen CH, Liang JD, et al. Hepatitis B and C viruses are not risks for pancreatic adenocarcinoma. *World J Gastroenterol*. 2014;20(17):5060-5065. doi:10.3748/wjg.v20.i17.5060

9. Hassan MM, Li D, El-Deeb AS, et al. Association Between Hepatitis B Virus and Pancreatic Cancer. *J Clin Oncol*. 2008;26(28):4557-4562. doi:10.1200/JCO.2008.17.3526

© 2025 Levinson RN et al. *JAMA Network Open*.
