# Supplementary material for: Pancreatic Ductal Adenocarcinoma After Hepatitis C Infection
Source: JAMA Netw Open. 2025 Nov 14;8(11):e2543701. doi: 10.1001/jamanetworkopen.2025.43701 (PMC12619103; doi:10.1001/jamanetworkopen.2025.43701)
Supplement: Supplement 2. — Data Sharing Statement [file jamanetwopen-e2543701-s002.pdf]

Data Sharing Statement

Levinson. Pancreatic Ductal Adenocarcinoma After Hepatitis C Infection. *JAMA Netw Open*. Published November 14, 2025. doi:10.1001/jamanetworkopen.2025.43701

Data
Data available: No

Additional Information
Explanation for why data not available: Given the regulations of the US department of Veterans Affairs and IRB ethics/privacy agreements, the analytic dataset used for this study are not permitted to leave the VA firewall without a data use agreement, a limitation that is also consistent with other studies using VA derived data. Upon request to the corresponding author, the analytical code will be made available.
